# The cost of a fall: using fall outcomes as a value-based care metric

**DOI:** 10.3389/fpubh.2026.1785717

**Published:** 2026-05-01

**Authors:** Jamar Haggans, Tobia Zanotto

**Affiliations:** 1 Department of Occupational Therapy Education, University of Kansas Medical Center, Kansas City, KS, United States; 2 Landon Center on Aging, University of Kansas Medical Center, Kansas City, KS, United States

**Keywords:** falls, occupational therapy, older adults, physical therapy, quality measures, value-based care

## Introduction

Falls are among the leading causes of injury and injury-related death for older adults, with 1 in 4 older adults reporting a fall each year (1). A Medicare and Medicaid claims analysis from 2008 to 2014 found that hospitalizations for traumatic injuries, of which falls are the most common cause, were more costly than hospitalizations for conditions such as congestive heart failure, pneumonia, stroke, and acute myocardial infarction (2). In a recent study, Haddad et al. found that non-fatal falls among older adults resulted in $80 billion of healthcare spending in 2020, an increase from $50 billion in 2015 (3, 4).

As the population of older adults increases and the healthcare system looks for ways to decrease spending while improving health outcomes, an increased focus on falls could be a viable solution. Although falls are a major contributing factor to preventable healthcare costs, fatalities, and injuries for older adults, there is limited representation of fall outcome measures in current value-based care (VBC) programs.

The purpose of this article—intended for policymakers, health system leaders, and healthcare administrators—is to examine the potential value of incorporating fall-related outcome measures into VBC frameworks for older adults. Integrating fall outcome measures into VBC models could benefit both patients and providers, while strengthening the foundation for improved safety, accountability, and clinical decision-making in the U.S. healthcare system.

## Value-based care

Healthcare spending in the U.S. has been steadily rising for several decades, although U.S. health outcomes such as life expectancy at birth, avoidable deaths, and infant and maternal deaths are worse compared to other developed nations (5). To address rising healthcare costs and poor outcomes, many payers have adopted alternative payment models (APM). VBC, also referred to as value-based payment (VBP), is an APM that incentivizes healthcare outcomes, tying payment to performance. The term *value* in VBC represents health outcomes in relation to the cost of achieving the outcome (6). VBC is a paradigm shift from the fee-for-service (FFS) model traditionally used for healthcare reimbursement in the U.S. In an FFS payment model, payment increases as providers provide more healthcare services, regardless of patient outcomes. VBC models align health outcomes to provider payments, incentivizing high-quality, coordinated care (7). The use of meaningful quality measures to evaluate performance and risk adjustment, the

practice of accounting for differences in client characteristics (e.g., disease severity, existing conditions, and social needs), are critical to sustainable VBC models.

Although the link to outcomes and payment is an essential part of VBC models, the models can differ greatly in their structure and risk shared by the organizations or providers. Literature on the efficacy of VBC models has been mixed. While many studies show participating in VBC models can have a positive impact on hospital readmissions, quality, spending, and utilization, others show only modest impacts (8, 21). However, experts have also stressed small shifts from the FFS model can be expected to have the least impact and may not be sufficient to produce large-scale change (9).

## Current use of falls measures in value-based care programs

Currently, fall-related outcome measures are minimally represented across VBC programs. Most existing measures focus on processes rather than outcomes.

For example, the *Falls: screening, risk-assessment, and plan of care to prevent future falls measure*, a process measure stewarded by the National Committee for Quality Assurance, is used in the Centers for Medicare and Medicaid Services (CMS) Merit-Based Incentive Payment System VBC program (10). While fall risk screening is an important first step, many experts argue the outcome of care, rather than processes, is most important when tying payment to performance (6).

A notable development occurred when CMS finalized that a quality measure evaluating the percentage of residents who experience a fall with a major injury would be added to the skilled nursing facility (SNF) value-based purchasing program, in fiscal year 2027 (11). This represents an important first step. Another opportunity to include fall outcomes (more specifically falls that lead to a major injury) is the home health value-based purchasing program (HHVBP). Home health agencies are already collecting this information; however, performance in the measure does not impact payment. Adding the falls with major injury measure in the HHVBP program represents an opportunity to align outcomes with payment, particularly as many older adults prefer to age in place.

## Rehabilitation professionals as implementation mechanisms in value-based care

Because VBC models reward organizations for improving outcomes and reducing avoidable utilization, rehabilitation professionals can function as core implementation mechanisms for fall-related quality measures. Occupational and physical therapy practitioners are commonly embedded within care settings that participate in VBC models. Their interventions directly influence metrics tied to financial incentives, including total cost of care, hospitalizations, emergency department use, and functional outcomes. This positions occupational and physical therapy practitioners as essential contributors to system-level performance improvement.

Occupational therapy practitioners are essential in identifying and treating barriers to safe engagement in meaningful activities that increase fall risk in older adults. Occupational therapy practitioners address factors such as functional cognition, activities of daily living (including functional mobility), health management and education, environmental modifications, and the use of assistive technology (12, 20). Evidence supports occupational therapy interventions such as individualized fall risk assessments, environmental modifications, fall risk education, and multisensory interventions to address behaviors (for clients with Alzheimer's disease and related major neurocognitive disorders) can be effective at reducing falls in older adults (13, 14). Other occupational therapy interventions that incorporate exercise programs with resistance training, dual tasking (i.e., combining physical and cognitive tasks simultaneously), and balance training have also been effective at reducing falls in older adults (13, 19).

Physical therapy practitioners also contribute significantly to reducing falls by addressing pain, strength, limitations in range of motion, fear of falling, and balance impairments that increase an older adult's risk of falls (15). Research shows a clear connection in fall reduction and physical therapy services including multicomponent exercise programs, progressive balance training, and gait adaptability training (16).

While occupational and physical therapy practitioners approach fall prevention from different clinical perspectives, both professions offer complementary and essential strategies for reducing falls and promoting safety among older adults. Integrating the clinical expertise of both occupational and physical therapy practitioners could significantly enhance the effectiveness of fall prevention programs by providing a more comprehensive person and function-centered approach.

Aligning front-line practice and system-level policy incentives to reduce falls positions rehabilitation professionals as essential drivers of fall-related performance improvement across the care continuum, balancing efforts to prevent falls with the need to maintain patient mobility.

## Final considerations

Falls represent a substantial and preventable cost burden to the U.S. healthcare system, as well as a major threat to older adults' health and independence. Despite the significant impact of falls on older adults, there remains a notable gap in research examining how fall outcome measures could enhance existing or future VBC models. The gap in research and policy presents an opportunity for policymakers and health services researchers.

Due to the proliferation of quality programs, and quality measures, there has been a recent focus to form a *universal foundation*. The universal foundation would align measures (between public and private entities and programs) based on conditions with the highest morbidity and mortality. Unfortunately, falls are not represented in the current version of the universal foundation (17). Relatedly, falls, even those without major injury, may represent an additional promising outcome measure, since falls can reduce confidence in performing activities of daily living and quality of life, regardless of whether an injury occurs.

Incorporating fall-related outcome measures into VBC models presents a powerful opportunity to align payment incentives with improved clinical outcomes. Such models would encourage providers to devote more resources (staff time, training, and interdisciplinary care planning) toward implementing effective fall prevention programs. Moreover, investing in fall prevention can drive improvements in other domains of care, such as functional mobility, activities of daily living, chronic condition management, and mental health (18).
